# Tin Oxide Nanowires Suppress Herpes Simplex Virus-1 Entry and Cell-to-Cell Membrane Fusion

**DOI:** 10.1371/journal.pone.0048147

**Published:** 2012-10-24

**Authors:** James Trigilio, Thessicar E. Antoine, Ingo Paulowicz, Yogendra K. Mishra, Rainer Adelung, Deepak Shukla

**Affiliations:** 1 Department of Ophthalmology & Visual Sciences, University of Illinois at Chicago, Chicago, Illinois, United States of America; 2 Department of Microbiology & Immunology, University of Illinois at Chicago, Chicago, Illinois, United States of America; 3 Department of Engineering, Institute for Materials Science, University of Kiel, Kiel, Germany; UC Irvine Medical Center, United States of America

## Abstract

The advent of nanotechnology has ushered in the use of modified nanoparticles as potential antiviral agents against diseases such as herpes simplex virus 1 and 2 (HSV-1) (HSV-2), human immunodeficiency virus (HIV), monkeypox virus, and hepatitis B virus. Here we describe the application of tin oxide (SnO_2_) nanowires as an effective treatment against HSV-1 infection. SnO_2_ nanowires work as a carrier of negatively charged structures that compete with HSV-1 attachment to cell bound heparan sulfate (HS), therefore inhibiting entry and subsequent cell-to-cell spread. This promising new approach can be developed into a novel form of broad-spectrum antiviral therapy especially since HS has been shown to serve as a cellular co-receptor for a number of other viruses as well, including the respiratory syncytial virus, adeno-associated virus type 2, and human papilloma virus.

## Introduction

There are seventy species of herpes viruses grouped into three subfamilies denoted as alpha, beta, and gamma, based on their biological and physical properties including cell tropism and genome organization. Herpes simplex virus type 1 (HSV-1), the α-subfamily prototype, is the primary cause of what is commonly known as “cold sores,” lesions of the mucosa of the mouth and lips. The seroprevalence of HSV-1 varies but increases with age and can reach up to 88% of the population by the age of 40 [1]. The most noteworthy feature of HSV-1 is its ability to establish latency after primary infection in host sensory neurons. This results in a lifetime of potential recurrences, usually at or near the original site of entry. In healthy individuals infections are often annoying but usually tolerable. In very mild cases many are even unaware of their status and spread the virus asymptomatically. However, in other instances the coarse nature of HSV-1 can turn into serious disease conditions such as ocular keratitis, retinitis, meningitis and encephalitis. In fact, HSV-1 is a leading cause of blindness and viral encephalitis in the developed world [2], and both are associated with severe morbidity [3]. Currently there is no cure or effective vaccine, only suppressive or episodic therapy with nucleoside analogues such as acyclovir, famciclovir or valtrex. All of these interfere with viral genome replication after cell penetration.

A more promising antiviral approach is to prevent the virus from entering the cell. For HSV-1 cell entry is a multi-step process mediated by viral envelope glycoproteins interacting with cell receptors, and fusion may occur at the plasma membrane or in endosomes [4]. Initially HSV-1 attaches to heparan sulfate proteoglycans (HSPG) at the host cell surface via viral envelope glycoproteins gB and gC. This likely causes a conformational change, and subsequently envelope glycoprotein gD binds to one of three alternative receptors: herpes virus entry mediator (HVEM), a member of the tumor necrosis factor (TNF) receptor family; Nectin-1, a member of the Nectin family of intercellular adhesion molecules; or 3 O sulfated heparan sulfate (3-OS-HS), a polysaccharide belonging to the heparan sulfate (HS) family. The three receptors are differently distributed in human cells and tissues. Receptor binding of gD, along with the help of three other glycoproteins (gB, gH, and gL), triggers fusion of the viral envelope with a cellular membrane [2]. Depending on the target cell, fusion takes place at the plasma membrane or in acidified endosomes.

Among the crucial entry steps the most promising target for an effective antiviral development is the initial interaction between the virus and cell in which the HSV-1 envelope glycoproteins gB and gC mediate attachment to cell surface HS [2]. This target is preferred because HS has the ability to bind numerous viruses and therefore offers the potential of a broad spectrum antiviral drug. In addition, interfering with this very first step in viral pathogenesis could have strong prophylactic effects as well.

Understanding this significance of HS in the infection process, along with recent advances in nanotechnology, spurred on the development of metal oxide based nanostructured compounds that mimic the viral binding ability of HS. One of these nanostructures, zinc oxide (ZnO), studied in our lab, has already shown this ability to compete for viral binding and suppress HSV-1 infection by such an emulating mechanism [5]. The cause of this attraction resides in the similar charge and shape comparable to the natural target (negatively charged HS attached to cell membrane filopodia). Nanostructures from other metal based materials have also shown similar antiviral properties such as silver nanoparticles capped with mercaptoethane sulfonate (Ag-MES) and gold nanoparticles capped with mercaptoethane sulfonate (Au-MES) [6], [7]. This mechanism is also shared with sulfated polysaccharides (dextran sulfate, pentosan polysulfate), and sulfated nonpolysaccharides (lignin sulfate, poly (sodium 4-styrene sulfonate), (T-PSS)) [7].

One of the latest nanostructures yet to be tested is tin oxide (SnO_2_) nanowires, the subject of this paper. In this study we investigated the potential of the negatively charged surface of SnO_2_ nanowires to bind and trap HSV-1 before entry into host cells. Here, through multiple biochemical and molecular based assays, we demonstrate the ability of SnO_2_ to significantly inhibit HSV-1 entry, replication, and cell-to-cell spread in naturally susceptible human corneal epithelial (HCE) cells.

## Results

### Synthesis of SnO_2_ Nanowires

**Figure 1:**
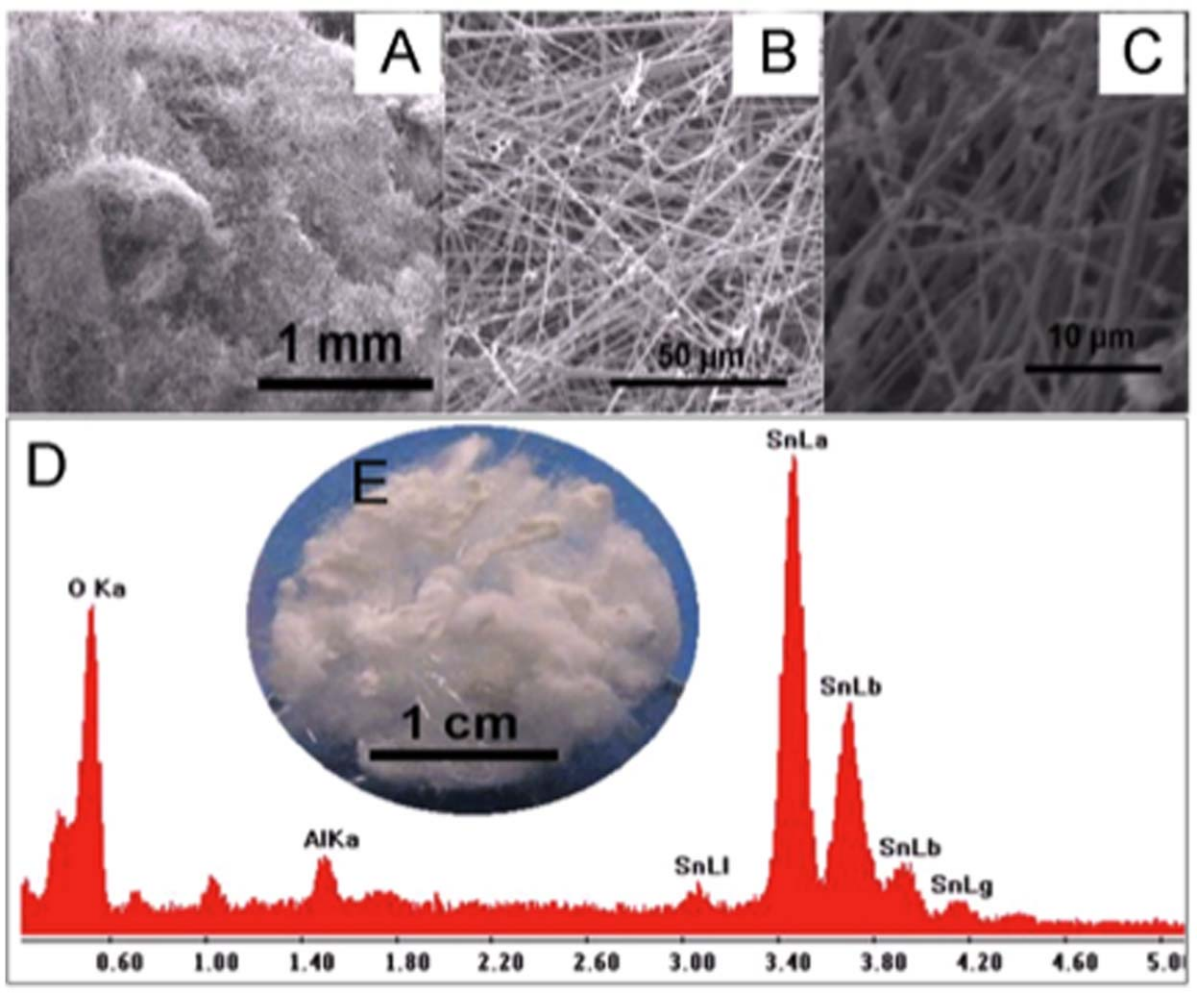
Scanning electron microscopy results of SnO_2_ nanowires synthesized by flame transport approach. A)–C): SEM images of SnO_2_ nanowires in increasing order of magnifications. D) Energy dispersive X-ray absorption (EDAX) spectrum showing the purity of SnO_2_ nanowires. The inset E) in D) is the digital camera image demonstrating the wire type fluffy structures of tin oxide.

SnO_2_ nanowires were produced by flame transport synthesis approach as described in the materials and methods section. Figures 1 A)–C) illustrate the 3D interconnected SnO_2_ network at micro- and submicro-scale, decorated with SnO_2_ nanocrystals. The lengths of these SnO_2_ wires vary from a few millimeters up to one centimeter or larger and their diameters range from hundreds of nm to the µm scale. A closer SEM view shows (Fig. 1C) that these wires exhibit decorations with very small crystals (50 to 100 nm in diameter) over the entire surface. Figure 1 D) shows an energy dispersive X-ray absorption (EDAX) spectrum which indicates that the synthesized product consists of pure SnO_2_ nano-microwires. The Al peak at 1.5 keV originates from the Al_2_O_3_ crucible that was used during synthesis. The inset 1 E) in 1 D) depicts the macroscopic view of the SnO_2_ snowflake type structure which was taken with a standard digital camera.

### SnO_2_ Nanowires have No Cytotoxic Effect on HCE Cells

**Figure 2:**
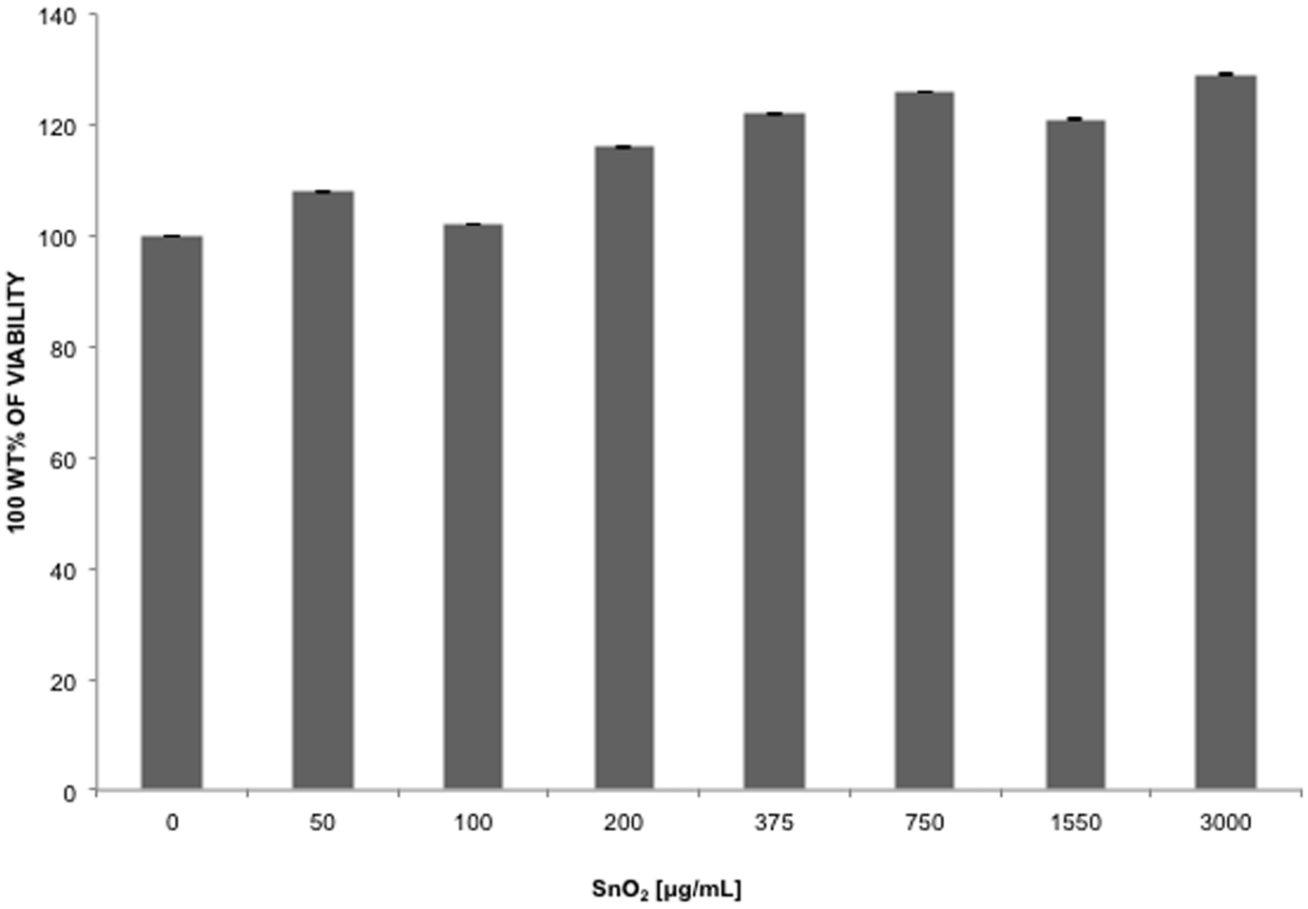
SnO_2_ cytotoxicity determination. To determine the effect of SnO_2_ nanowires on cell viability a cytotoxicity assay was performed. HCE were treated for 24 hours in the presence of SnO_2_. Cell viability was evaluated by a chromogenic kit (CellTiter Aqueous96; Promega, Madison, WI, USA) and colorimetric detections were performed using a mircroplate ELISA reader (Spectra Max 190). Results are expressed as 100% wild type (WT) viability where they represent the percent corrected absorbance after subtracting the background absorbance relative to untreated cells (0 µg/ml).

The cytotoxicity of SnO_2_ nanowires were assessed in HCE cells by an MTS cell proliferation assay and later confirmed by a trypan blue cell counting assay. As seen in Figure 2, no dosage dependent cytotoxicity was observed, even at the highest dosage of 3000 µg/ml. Unlike ZnO treatment in HCE cells that resulted in a 50%–70% decrease in viability at a concentration 1 mg/ml [5], SnO_2_ treated HCE cells ability to proliferate was not affected by treatment conditions. To confirm the results of the cell viability assay a trypan blue cell staining assay was carried out. As observed in the cell viability assay SnO_2_ treatment of 3000, 1500, 750, 375, 187, 93, or 47 µg/ml had no effect on the viability of cells 24 hours post treatment (data not shown).

### SnO_2_ Nanowires Block HSV-1 Entry into Naturally Susceptible Cells

**Figure 3:**
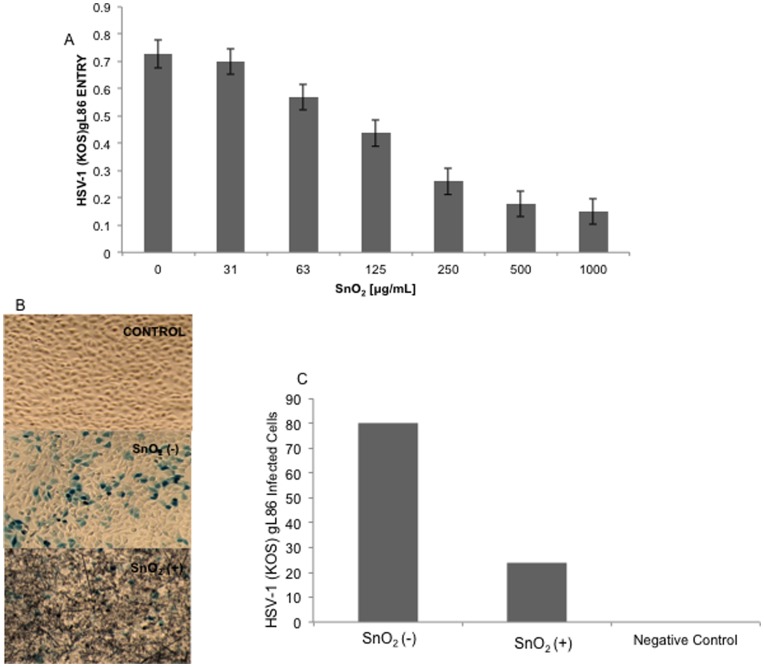
SnO_2_ inhibits HSV-1 entry into HCE cells. HCE cells were mock treated or treated with SnO_2_ and exposed to HSV-1 at an MOI of 10 for 6 hours. A) After 6 hours of infection cells were washed, permeabilized and incubated with ONPG substrate for quantification of β-galactosidase activity from the viral genome. A dosage dependent decrease in entry was noted in cells as minimal entry occurred. B) X-gal staining of HCE cells. HCE cells grown in a 6-well plated were pretreated with SnO_2_ before being challenged with HSV-1 for 6 hours. Cells were washed with PBS, fixed, permeabilized and incubated with X-gal, yielding blue cells. Infected cells were imaged at 10× objective using Zeiss Axiovert microscope. C) The average number of infected cells in SnO_2_ treated cells is significantly lower than mock treated cells.

To determine the antiviral properties of SnO_2_ nanowires against HSV-1 entry, a confluent monolayer of HCE cells were cultured in a 96-well plate, treated with serial dilutions of SnO_2_ and infected with recombinant HSV-1(KOS) gL86 virus which expresses beta-galactosidase within its genome. Untreated SnO_2_ HCE cells were used as a positive control. Entry of HSV-1 was measured 6 hours post infection using an ONPG colorimetric assay [8]. As shown in Figure 3A, SnO_2_ nanowires inhibited entry in a dosage dependent manner with maximum viral entry occurring at the lowest concentration (31 µg/ml) of SnO_2_ treatment. At higher concentrations of SnO_2_ treatment HSV-1 entry was significantly decreased. HSV-1 entry in cells treated at a concentration of 500 µg/ml and 1000 µg/ml was 5 times lower than untreated cell HCE cells. These results, together with results from our cell viability assay, show that we can obtain a maximum inhibition of entry at a concentration of 500 and 1000 µg/ml without compromising the health of the cells. Due to only a 7% difference in viral entry at concentrations of 500 µg/ml and 1000 µg/ml, 500 µg/ml was chosen as the treatment dose for all subsequent experiments.

Next an X-gal entry assay was utilized to further confirm the efficacy of SnO_2_ nanowires against HSV-1 entry. HCE cells were grown in a 6-well plate and treated with SnO_2_ and a beta-galactosidase-encoding recombinant virus, (along with +/− control wells). In the presence of X-gal substrate, cells that had been virally infected obtained a blue color, allowing visual analysis of infected cells (Figure 3B). Uninfected cells display no color change (Negative Control). The number of virally infected cells within SnO_2_ nanowire treated cells was significantly lower than cells that had not undergone SnO_2_ treatment (Figure 3C). The numerical results of Figure 3C were obtained from the average of six samples in each condition, suggesting that the susceptibility of HCE to HSV-1 infection decreases in the presence of SnO_2_, thus protecting cells from the virus.

### SnO_2_ Nanowire Treatment Reduces Viral Replication, Plaque Formation and Plaque Size

**Figure 4:**
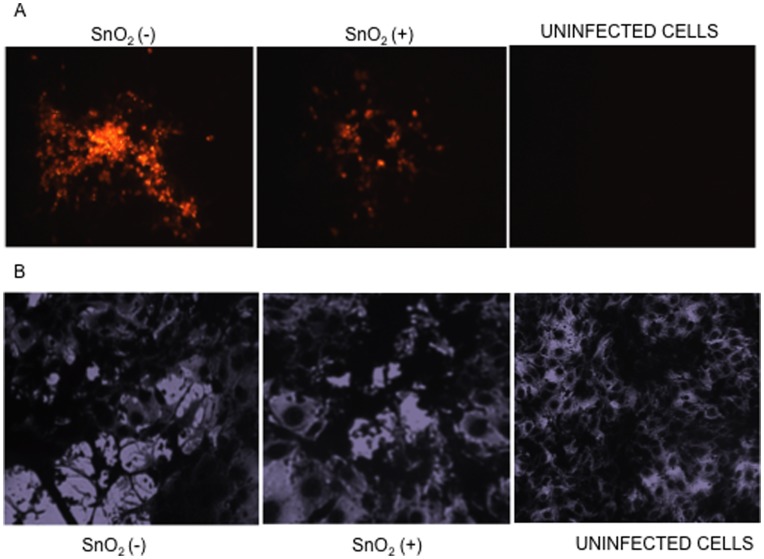
SnO_2_ Inhibits cell-to-cell spread and plaque formation in HCE cells. A) Confluent monolayers of HCE cells were infected with HSV-1 (KOS) K26RFP and viral replication and spread were imaged 72 hours post infection. The effect of SnO_2_ on viral spread was assayed through the measurement of infected cell clusters and the intensity of RFP emission. B) In conjugation with the infectious spread assay, a plaque assay was performed to evaluate the SnO_2_ effect on viral transmission. UV treated SnO_2_ was added to cells prior to a 2 hour incubation with HSV-1(KOS). Following the 2-hour absorption phase virus inoculum was removed and cells were overlaid with methylcellulose. 3-days post infection cells were fixed with methanol at room temperature for 20 minutes and strained with crystal violet. Images were taken with a Zeiss Axiovert 200 microscope using a 10× objective.

Since treatment with SnO_2_ nanowires resulted in decreased viral entry, we hypothesized that there should be a net reduction in viral replication as well because a significantly low number of virus particles can enter cells in the presence of SnO_2_. In order to visually analyze how SnO_2_ treatment effected viral entry which in turn reduced replication, SnO_2_ treated HCE cells were infected with HSV-1 (KOS)K26RFP virus. Fluorescence microscopy was used to visualize the production of virons in cells several days post infection. As seen in Figure 4A, RFP intensity (red color representative of virus production) in SnO_2_ treated cell was much lower than untreated cells. Under normal infection conditions, the virus spreads naturally to neighboring cells, however we observed that in SnO_2_ treated cells many neighboring cells were uninfected black in comparison to mock treated cells which displayed a higher RFP intensity, which is representative of more virus production.

To further assess the effect of SnO_2_ nanowires on entry and its resultant effect on viral replication, a plaque-forming assay was performed. The observation of plaques, the central clearing of cells as the virus spreads outward [9], has been one of the key indications of cell to cell viral spread. Since SnO_2_ treatment decreased replication, we further investigated whether SnO_2_ treatment affected the lateral transmission of HSV-1 in order to form plaques. To determine the SnO_2_ nanowire’s effect on plaque formation, confluent monolayers of HCE cells were treated with SnO_2_ (or mock treated) and infected with HSV-1 (KOS) virus for 2 hours, after which SnO_2_ and inoculums were removed and cells overlaid with methylcellulose. Several days post infection cells were fixed and stained and plaques were counted. As seen in Figure 4B, HCE cells pretreated with SnO_2_ produced plaques that were 75% smaller than mock treated cells. Analysis also revealed that SnO_2_ treatment resulted in 40% less plaque formation. These results taken together suggest that productive replication and viral spread is decreased when cells are treated with SnO_2_ nanowires.

### SnO_2_ Nanowire Treatment Inhibits HSV-1 Glycoprotein Mediated Cell-to-cell Fusion

**Figure 5:**
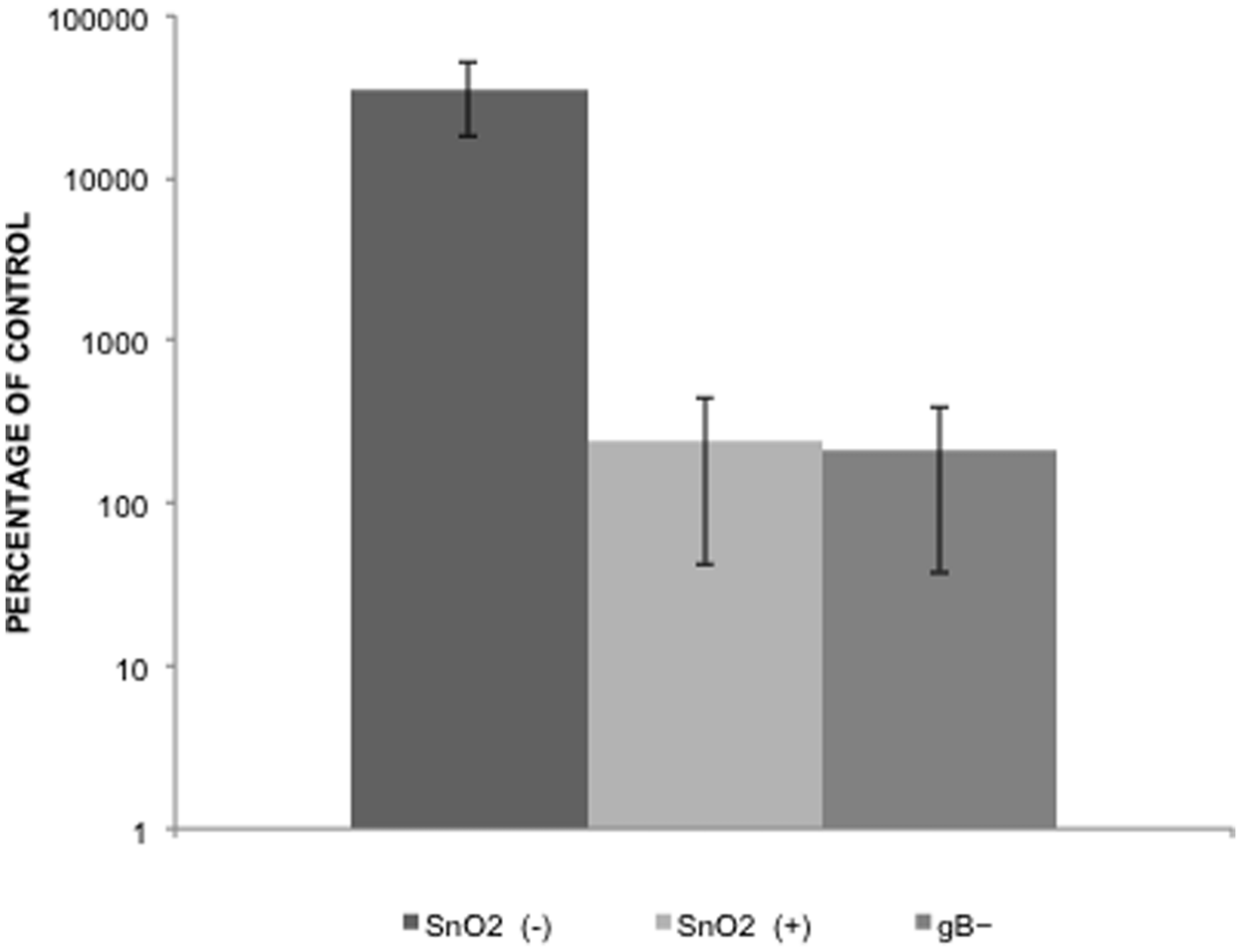
SnO_2_ treatment reduces glycoprotein mediated cell-to-cell fusion. Two populations of cells were generated to determine the effect of SnO_2_ treatment on cell fusion. Effector cells were transfected with plasmids gB, gD, gH, gL and T7. Target cells were transfected with gD, receptor Nectin-1 and a luciferase expressing plasmid under the control of a T7 promoter. Target and Effector cells were mixed together at a 1∶1 ratio. Luciferase activity was determined in the presence of firefly luciferase, allowing the measurement of relative light units (RLU). CHO-K1 cells were either mock treated or treated with SnO_2_. As a negative control, effector cells lacking gB were mixed with the target cells.

An efficient mode of transmission that the virus uses to infect neighboring cells requires cell-to-cell fusion [2]. Through this process the virus spreads across the junctions between the membranes of adjacent cells and causes infected cells to aggregate into clumps [10]. By this mode of transmission HSV increases pathogenesis while evading detection of the immune system. Cell-to-cell transmission of HSV-1 is highly dependent on glycoproteins gD, gB, gH, and gL, thus viruses deficient in any of the four glycoproteins are defective in their ability to spread. Since SnO_2_ treatment resulted in a decrease of viral entry and replication, we decided to further investigate how SnO_2_ treatment affected glycoprotein mediated cell-to-cell fusion. Figure 5 shows a great impairment of glycoprotein mediated cell-to-cell fusion in cells treated with SnO_2_ nanowires. The 99% decrease found amongst treated cells was comparable to the level of fusion in the cells lacking glycoprotein B (Figure 5).

### Fluorescently-labeled SnO_2_ Nanowires Bind HSV-1(KOS) K26GFP

**Figure 6:**
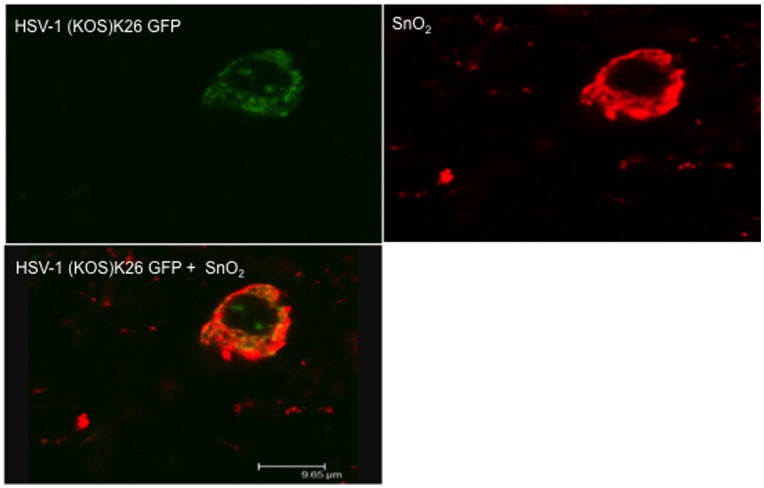
SnO_2_ exhibits HSV-1 binding ability. A binding assay was preformed to determine the interactions of SnO_2_ with K26 GFP virus. A SnO_2_ solution was placed into an Eppendorf tube and stained with Rhodamine phalloidin [red](Invitrogen) for 20 minutes, washed with PBS, and incubated with HSV-1 (KOS) K26GFP for 2 hours at 37°C. Leica Confocal microscopy software was utilized to identify co-localization of SnO_2_ with HSV-1.

HSV entry is a multistep process that can be grouped into two phases, viral attachment and viral fusion. The attachment phase initiates the virus’s first contact with the host cell through the binding of viral glycoproteins to heparan sulfate proteoglycans (HSPG) [11]. Through the interactions of gB and gC with heparan sulfate side chains the virus is enabled to bind and further contact its cell surface receptors [12]. Presently, the function of polyanionic compounds as anti-HSV agents is being extensively explored as these molecules compete with HS for viral binding. As a result of the slight negative charge nanostructures such as ZnO, Au and Ag have been found to directly interact with HSV, thereby inhibiting viral pathogenesis. To determine whether SnO_2_ directly interacts with HSV-1 a viral binding assay was performed. We rationalized that the slight negative charge generated post irradiation would attract virus particles to the SnO_2_ nanowire surface at a high affinity. To visualize this effect SnO_2_ was stained with Rhodamine Phalloidin [13] (Invitrogen) and incubated with HSV-1 (KOS) K26GFP for 2 hours. Confocal microscopy results revealed the co-localization of SnO_2_ with HSV-1 (Figure 6), thus supporting the hypothesis that SnO_2_ nanowires exhibit a viral trapping ability which neutralizes HSV-1, limiting viral entry, replication and spread.

## Discussion

So far herpes simplex virus type 1 has eluded a cure. Obstacles such as latency in non-dividing neurons, emergence of resistant strains or adverse side effects have contributed to this difficulty. Although there are many anti HSV-1 drugs available to treat symptoms, they do not eliminate the virus or stop the spread of existing virions, and severe complications such as blindness and encephalitis are still possible. This is especially true in neonates and immunocompromised patients. Drug resistance has also been reported in the latter. One way the effects of current therapies against HSV can be enhanced is by targeting multiple steps in HSV pathogenesis. Multi-targeted therapy against HSV has not been possible due to lack of well-studied new targets. Therefore, the development of alternate antiviral agents needs to be a top priority for this highly contagious global disease. Targeting entry and more specifically HS attachment during viral pathogenesis with SnO_2_ appears to fulfill this requirement and shows the promise of providing great benefits in multi-drug therapy against HSV. Since attachment to cell surface HS is the first step in the infection process for many viruses, including HSV-1, it should naturally be an attractive model for an antiviral defense mechanism. And of course, blocking entry has the added advantage of minimizing or eliminating all of the following steps in the infectious cycle.

In these experiments SnO_2_ nanowires have shown an ability to compete for virus at the attachment step by acting like the natural target (HS), similar to what ZnO, Ag-MES, and Au-MES do. Since the amount of virus that enters the cell has a direct relationship with disease severity and reactivation rates, minimizing or blocking the viral load with SnO_2_ is certainly expected to substantially reduce the distressful and sometimes agonizing results of an untreated infection.

In keeping with its emerging biological applications [14] and relatively non-toxic nature under in vitro conditions (Figure 2), the SnO_2_ nanowires used here were found to be an effective inhibitor of viral entry and cell-to-cell spread. The concentrations of SnO_2_ used in our study were well below any significant cytotoxic levels. The results on average showed a 75% reduction in cell entry, 77% smaller plaques or infected cell clusters and over a 99% drop in cell-to-cell fusion. Reduced entry also translated into reduced replication and spread to other cells. We consider these results very promising for any future development of SnO_2_ nanowires as anti-HSV agents, especially as new and effective prophylactic agents. In this regard it will also be very interesting to test their efficacies against other viruses and microbes that use HS for attachment to cells [15].

Although this antiviral mechanism is not unique to SnO_2_ nanowires it might be more cost effective and tolerable as compared to the other nanoparticles previously mentioned. Future considerations should be made to rank the effectiveness of these antivirals in-vitro, followed by in-vivo studies and formulation trails. Combination therapy could also improve the results as SnO_2_ and ZnO used together could potentially decrease the cytotoxicity while enhancing the efficacy of the treatment. It might also be possible to present these compounds as virus trappers that stimulate immune response while providing protection from virus infection as microbicides. The combined effect would lead to improved viral clearance and overall antiviral effectiveness. In conclusion, SnO_2_ nanowires show the novel promise to prevent and/or reduce the complications associated with HSV infection and represent a new class of anti-viral agents that require further testing in animal models and against other pathogenic viruses.

## Materials and Methods

### Preparation and Characterization of SnO_2_ Nanowires

SnO_2_ micro−/nanowires were produced by employing the Flame Transport Synthesis (FTS) [16], [17]. 20 g of Polyvinylbutyrale (PVB), (Kuraray, Mowital) was dissolved in 40 g Ethanol (Carl Roth, 99.8% denatured with 1% MEK) under intense stirring. To the obtained viscous solution 10 g of Sn powder (AlfaAesar, 99.9%, 1–5 µm) was added whilst stirring. 10 g of the honey-like dispersion was put into a crucible and heated in a muffle-furnace at 950°C for 4 hours. The product had a white color and cotton like appearance. The morphological evolutions and chemical purity of synthesized SnO_2_ nanowires were investigated inside a scanning electron microscope (SEM) machine, Philips XL 30 (LaB_6_ Cathode, acceleration voltage15 kV) followed by an energy dispersive X-ray analysis.

All experiments used SnO_2_ nanowires irradiated for 1 hour with ultraviolet light (UV) in a petri dish or a sterile 50 ml polypropylene tube. Applying UV light before treatment increased its negative charge, further attracting the HSV-1 virus. UV treated SnO_2_ was brought into suspension, then to increase its solubility and homogeneity it was sonicated twice for 15 seconds before use in experiments.

### Cell Culture, Plasmids, and Virus

Human corneal epithelial cell line (RCB1835 HCE-T) was provided by Dr. Kozabauro Hayashi (National Eye Institute, Bethesda) [18]. HCE cells were passaged in Minimum Essential Medium (MEM) (Gibco/BRL, Carlsbad, CA, USA) supplemented with 10% fetal bovine serum (FBS) and penicillin and streptomycin (P/S) (Sigma). HeLa cells were provided by B.P.Prabhakar (University of Illinois at Chicago). HeLa cells were passaged in Dulbecco’s modified Eagle’s medium (DMEM) (Gibco/BRL, Carlsbad, CA, USA) supplemented with 10% FBS and P/S. Chinese hamster ovary (CHO-K1) cells were provided by P.G. Spear (Northwestern University). CHO-K1 cells were passaged in Ham’s F12 medium (Gibco/BRL, Carlsbad, CA,USA) supplemented with 10% FBS and P/S.

Plasmids expressing HSV-1 glycoproteins pPEP98 (gB), pPEP99 (gD), pPEP100 (gH), and pPEP101 (gL) were used in this study [19]. Plasmid pT7EMCLuc that expresses the firefly luciferase gene under the control of a T7 promoter and plasmid pCAGT7 that expresses T7 RNA were also used [20].

P.G.Spear (Northwestern University) provided wild type HSV-1 (KOS) strain and recombinant HSV-1(KOS)gL86 strain [8]. HSV-1 (KOS) K26RFP and HSV-1 (KOS) K26GFP virus strains were provided by P. Desai (Johns Hopkins University). Jellyfish green fluorescent protein was fused in framed with the UL35 open reading frame generating K26GFP virus whose capsids express GFP [21]. Virus stocks were propagated and tittered on Vero cells and stored at −80°C.

### Cytotoxicity Assay

To determine the effect of SnO_2_ nanowires on the viability of HCE cells an MTS cytotoxicity assay was performed after 24 hours of SnO_2_ treatment. Briefly, HCE cells were seeded at a density of 2×10^4^ in a 96-well plate and incubated until confluent. SnO_2_ was then brought into suspension in MEM media at concentrations of [3000, 1500, 750, 375, 187, 93, 47, or 0] µg/ml and added to the appropriate wells. 24 hours later the cell viability was analyzed by a chromogenic kit (CellTiter Aqueous96; Promega, Madison, WI, USA). Colorimetric detection was measured by a micro-pate reader (TECAN GENious Pro) at 492 nm. Results are represented as 100% wild type viability.

### Viral Entry Assays

A standard entry assay was performed as described previously [8]. Briefly, HCE cells were seeded at a density of 2×10^4^ in a 96-well plate. Upon confluency cells were both treated with dilutions of SnO_2_ at [1000, 500, 250, 125, 62, 31, 0] µg/ml and infected with beta-galactosidase expressing recombinant virus HSV-1 (KOS)gL86 at a multiplicity of infection equal to 10 (MOI = 10) for 6 hours at 37°C. After 6 hours cells were washed with PBS and soluble substrate *o-*nitrophenyl-beta-D-galactopyranoside (ONPG ImmunoPure, PIERCE,) was added. Enzymatic activity was measured by a micro-pate reader (TECAN GENious Pro) at 405 nm.

An X-gal staining entry assay was also performed to confirm the effect of SnO_2_ treatment on HSV-1 entry as described previously [11]. Briefly, HCE cells were grown in a 6-well plate until confluent and then treated (or mock treated) with 500 µg/ml of SnO_2_ and infected with HSV-1 (KOS)gL86 reporter virus (MOI = 10). 6 hours post infection cells were washed with PBS and fixed with 2% formaldehyde and 0.2% glutaradehyde at room temperature for 15 minutes. Cells were washed with PBS and permeabilized with 2 mM MgCl_2_, 0.01% deoxycholate and 0.02% Nonidet NP-40 for 15 minutes. After washing cells with PBS cells were treated with ferricyanide buffer containing beta-galactosidase substrate X-gal. Cells were assessed by capturing images of blue cells at a 10× objective (Zeiss Axiovert 200).

### Plaque Assay

A monolayer of HCE cells were seeded in a 6-well plate at a density of 3×10^6^ cells per well. Upon confluency cells were treated (or mock treated) with 500 ug/ml of SnO_2_ nanowires and infected at a MOI of 0.001 with HSV-1 KOS virus strain. 2 hours post infection inoculums were removed and methylcellulose (Sigma) was added to the wells. Cells were then incubated at 37°C for 3 days. At the end of the incubation cells were fixed with methanol for 20 minutes at room temperature and stained with crystal violet. Plaques were counted and imaged at 10× objective (Zeiss Axiovert 200).

### HSV-1(KOS) K26RFP Virus Spread Assay

A virus-spread assay was performed as described previously [22]. Briefly, a monolayer of HCE cells were treated (or mock treated) with 500 µg/ml of SnO_2_ and challenged with HSV-1(KOS) K26RFP virus strain at a MOI of 0.001. 2 hours post infection inoculum was removed. Cells were washed once with PBS and overlaid with methylcellulose (Sigma) MEM medium. 72 hours post infection the spread of HSV-1(KOS) K26RPF amongst HCE cells was assessed by capturing images of the red virally infected cell clusters using a 10× objective (Zeiss Axiovert 200).

### Virus-free Cell-to-cell Fusion Assay

A standard virus free cell-to-cell fusion assay was performed as described previously [20], [21]. To create the fusion process in vitro CHO-K1 cells were split into two populations: target cells, and effector cells [23]–[25]. The target cell population was transfected with 1.0 µg of gD receptor (Nectin-1) and 0.5 µg of the plasmid expressing the luciferase gene. The effector cell population was transfected with 0.5 µg each of HSV-1 glycoproteins pPEP98 (gB), pPEP99 (gD), pPEP100 (gH), and pPEP101 (gL), along with plasmid pCalifGT7, which expresses T7 RNA polymerase. Fusion between both populations of cells allows the T7 polymerase to bind its promoter thus activating the synthesis of the luciferase gene. Following addition of the substrate, (D-luciferin), the extent of fusion between target and effector cells can be analyzed. At 24 hours post transfection effector and target cells were either mock treated or treated with 500 µg/ml of SnO_2_ nanowires during their 1∶1 co-culture in a 24-well dish. 24 hours post mixing effector and target cell luciferase gene expression resulting from fusion was measured using a reporter lysis assay (Promega).

### Mixing of Fluorescent-labeled SnO_2_ with GFP-tagged HSV-1

The binding ability of SnO_2_ nanowires to HSV-1(KOS) K26GFP was determined by mixing fluorescently labeled SnO_2_ with HSV-1 (KOS) K26GFP. First 500 µl of PBS was added to 0.5 mg of SnO_2_ and sonicated for solubility. 20 µl of this SnO_2_ solution was then added to 20 µl of 10 nm Rhodamine phallodin [13] (Invitrogen) and incubated at room temperature for 30 minutes. At the end of incubation the SnO_2_ particles were washed by centrifuged twice with PBS to remove unbound dye. At the completion of the wash step the fluorescently labeled SnO_2_ nanowire pellet was resuspended in 400 µl of OptiMEM media. HSV-1 (KOS) K26GFP virus was then added to 200 µl of the labeled SnO_2_ nanowire solution and placed in a 35 mm glass bottom dish and incubated for 2 hours at 37°C. A second dish with the other 200 µl of labeled SnO_2_ was used as a control. At the end of incubation the fluorescently labeled SnO_2_ nanowires and HSV-1(KOS) K26GFP (as well as the control dish) were imaged on a confocal microscope (Lecia DMIRE2) equipped with a camera (Lecia TCSSP2).
